# Loss-of-Function of *Constitutive Expresser of Pathogenesis Related Genes5* Affects Potassium Homeostasis in *Arabidopsis thaliana*

**DOI:** 10.1371/journal.pone.0026360

**Published:** 2011-10-27

**Authors:** Monica Borghi, Ana Rus, David E. Salt

**Affiliations:** Department of Horticulture and Landscape Architecture, Purdue University, West Lafayette, Indiana, United States of America; Ohio State University, United States of America

## Abstract

Here, we demonstrate that the reduction in leaf K^+^ observed in a mutant previously identified in an ionomic screen of fast neutron mutagenized *Arabidopsis thaliana* is caused by a loss-of-function allele of *CPR5,* which we name *cpr5-3*. This observation establishes low leaf K^+^ as a new phenotype for loss-of-function alleles of *CPR5*. We investigate the factors affecting this low leaf K^+^ in *cpr5* using double mutants defective in salicylic acid (SA) and jasmonic acid (JA) signalling, and by gene expression analysis of various channels and transporters. Reciprocal grafting between *cpr5* and Col-0 was used to determine the relative importance of the shoot and root in causing the low leaf K^+^ phenotype of *cpr5*. Our data show that loss-of-function of *CPR5* in shoots primarily determines the low leaf K^+^ phenotype of *cpr5*, though the roots also contribute to a lesser degree. The low leaf K^+^ phenotype of *cpr5* is independent of the elevated SA and JA known to occur in *cpr5*. In *cpr5* expression of genes encoding various *Cyclic Nucleotide Gated Channels (CNGCs)* are uniquely elevated in leaves. Further, expression of *HAK5*, encoding the high affinity K^+^ uptake transporter, is reduced in roots of *cpr5* grown with high or low K^+^ supply. We suggest a model in which low leaf K^+^ in *cpr5* is driven primarily by enhanced shoot-to-root K^+^ export caused by a constitutive activation of the expression of various *CNGCs*. This activation may enhance K^+^ efflux, either indirectly via enhanced cytosolic Ca^2+^ and/or directly by increased K^+^ transport activity. Enhanced shoot-to-root K^+^ export may also cause the reduced expression of *HAK5* observed in roots of *cpr5*, leading to a reduction in uptake of K^+^. All ionomic data presented is publically available at www.ionomicshub.org.

## Introduction

Potassium (K^+^) is a macronutrient essential for normal plant growth and development. It participates in numerous physiological processes including regulation of enzyme activity, cell expansion, stomata movement and defence towards pathogens [1], [2]. Numerous mechanisms are known to be involved in K^+^ homeostasis, and many K^+^ channels and transporters have been identified. In plants K^+^ is highly mobile. Its movement into the xylem is driven by transpiration, and in the phloem by the specific requirements of tissues and organs [3], [4]. Environmental conditions also regulate the movement of K^+^. For example, under drought stress the concentration of K^+^ in the xylem decreases, possibly because of the closure of stomatal pores induced by abscisic acid (ABA) [5].

The effects of low K^+^ have been investigated in some depth, and it has emerged that jasmonic acid (JA) plays a central role in the response of plants to K^+^ shortage [6], [7]. Nevertheless, whether the application of K^+^ is beneficial or not in conferring resistance towards pathogens is controversial [8]. At the molecular level the role of K^+^ is clearer. Plant-pathogen interactions cause an increase in cytosolic Ca^2+^ triggering anion channel activation, plasma membrane depolarization, activation of *K^+^* permeable efflux channels leading to enhanced K^+^ efflux and the initiation of the hypersensitive response (HR) [9], [10].

In this study, we describe the characterization of an *A. thaliana* mutant with a 10–30% reduction in leaf K^+^ which was previous identified in an ionomic screen of fast neutron mutagenized plants [11]. Genetic analysis revealed this mutant to be a new null allele of *CPR5*, a gene originally identified in two independent screens for altered response to pathogens [12], [13]. *cpr5* has a high content of salicylic acid (SA) and shows constitutive expression of pathogenesis related genes (*PR*), as well as plant defensin *PDF1.2* which is a marker of the JA-dependent pathway. Therefore, it has been suggested the *CPR5* is a negative regulator of local defence response to pathogens [14]. *CPR5* is also implicated in cell senescence [15]–[17], cell proliferation and trichome development [18], cell wall biogenesis [19] redox balance [20], and water relations via enhanced ABA sensitivity [21]. Here, we show that *CPR5* is also associated with K^+^ homeostasis possibly via modulation of expression of various *CNGCs* and *HAK5*.

## Materials and Methods

### Plant growth and mutant screening

Fast neutron-mutagenized M2 *A. thaliana* seeds were purchased from Lehle Seeds (Round Rock, TX) and plants screened for their leaf elemental profile by ICP-MS [11]. Seeds of Col-0 (CS6000) and *cpr5-2* (CS3770) were provided by the Arabidopsis Biological Resource Center (The Ohio State University).

For non axenic conditions, plants were grown in pots containing moist soil (Scotts Potting Medium, Scotts-Sierra Horticultural Products Company, Marysville, OH) in a climate-controlled room (temperature 19–22°C, day-night; humidity 60%; photoperiod 10–14 hours light-dark; light intensity 100±10 µmol m^−2^ sec^−1^) and bottom watered at regular intervals with a solution containing 0.25× Hoagland's macro and micronutrients [11].

For plants grown in axenic conditions, surface sterilized seeds were stratified at 4°C in the dark for five days before sowing. To measure the expression of *HAK5* and *AKT1* plants were grown for 2 weeks on solidified medium containing 1/20^th^ MS salts accordingly to Cheong et al., [22] and containing 20 mM or 100 µM of KCl, and in a second experiment on a minimal medium without NH_4_^+^ [23] with 10 g L^−1^ UltraPure sucrose (Sigma) and solidified with 10 g L^−1^ pure agarose (Molecular Biology Grade, Research Products International Corp.), which contains negligible amounts of K^+^ (approximately 8 µg Kg^−1^ as determined by ICP-MS analysis). K^+^ was added as KCl at the final concentration of 10, 50 and 100 µM and the pH adjusted to 5.8 with Ca(OH)_2_. K^+^ content in root and shoot was measured in plants grown for 2 weeks on solidified medium containing 1/20^th^ MS salts accordingly to Cheong *et al.,* [22] and containing 20 mM of KCl.

### Genetic analysis

*cpr5-3* was outcrossed to *A. thaliana Landsberg erecta* (L*er*-0) accession, F1 seeds were planted on 0.5× MS medium solidified with 10 g L^−1^ of agar and seedlings visually screened for hyponastic and early yellowing cotyledons. All seedlings of F1 generation looked wild-type and were transferred to soil and grown to produce F2 seeds. F2 plants grown on soil were visually selected for small size and yellow early senescing leaves and used to map the mutation with a positional cloning approach with single strand length polymorphism (SSLP) markers.

### Quantitative real-time PCR

Total RNA was extracted with the Qiagen RNeasyPlant Mini Kit (http://www.qiagen.com) from five weeks old plants grown on soil or from two week old plants grown on plates. DNase digestion on column was performed to eliminate possible contamination with DNA. Two micrograms of total RNA were used as a template to synthesize first-strand cDNA with SuperScript VILO cDNA Synthesis Kit (InvitrogenLife Technologies, http://www.invitrogen.com). Quantitative real-time PCR was performed with the SYBR Green reagent mix in a StepOnePlus instrument according to the manufacturer's instructions (Applied Biosystems, California, USA). The expression of K^+^ channel/transporter genes was detected with the following set of primers: *HAK5*, 147 (5′-TGCTGATCTAGGTCACTTCAGTGTT-3′) and 148 (5′-AAAGCAGGATATGCGACACATG-3′); *AKT1*, 170 (5′-TCTAAATTGTGTTCTTCTTCTGTTGGA-3′) and 171 (5′-CCTTCCGCGTCTCTGCAA-3′); *CNGC*19, 137 (5′-TTCTCACTTGGTGCCTCTCTTCT-3′) and 138 (5′-AATCCCTTTGGTGGCATCTTT-3′); *CNGC10*, 139 (5′-TCATCATTGATCTACTCTCTATCCTTCCT-3′) and 140 (5′-TGGTTGACGCTTGGAATAACG-3′); *CNGC12*, 156 (5′-CAACGAACATTCAGGTTATACTCACA-3′) and 157 (5′-GCCGCTTGAATGAAGAATGC-3′); *CNGC20*, 160 (5′-TGCCTCGAACGCTCTTCTG-3′) and 161 (5′-CCTTTGATGGCATCCTTATCCT-3′); *CNGC11*, 196 (5′-GATAAAAACATGAATCTTCAGAGGAGAA-3′) and 197 (5′-CTAACACTTTTCAATTTTCCATCAACTC-3′). As an internal reference the expression of *PP2A* (At1g13320) was used since it showed stable expression throughout the experimental series of development, shoot and root abiotic stress, hormones, nutrient stress, light and biotic stress [24], all of which are affected by loss-of-function of *CPR5*. In addition to *PP2A* the expression of *HAK5* and *AKT1* was also normalized to *UBQ10* (At4g05320). *UBQ10* also shows stable expression in *cpr5*. The average value from real-time PCR measurements from at least three independent biological replicates was used to evaluate transcript abundance. Biological replicates were composed of tissues harvested from between 5 and 10 plants for analysis of shoot tissue, and between 25 and 30 plants for analysis of root tissue, from plants grown on plates. For plants grown in soil 2–3 leaves from at least 3 individual plants were used. Steady state mRNA levels were calculated relative to a reference gene and presented based on the 2^−Δ*C*t^ method [25].

### Determination of the elemental content of plant tissues

Plants grown in soil were non-destructively sampled by removing 1–2 leaves (approximately 3 mg dry weight), rinsed with 18 MΩ water, placed into Pyrex digestion tubes and dried at 92°C for 20 hours. Alternatively, shoots and roots were harvested from plants grown on 1/20^th^ MS medium modified accordingly to Cheong et al. [22] as previously described. After cooling, all samples were digested with 0.7 mL concentrated nitric acid (OmniTrace, VWR) and diluted to 6.0 mL with 18 MΩ water. Acid used for digestion was spiked with gallium (Ga) to act as an internal standard to control for errors in dilution, variations in sample introduction, and plasma stability in the ICP-MS instrument. Sample sets also contained analytical blanks, standard reference material (NIST SRM 1547) digested in the same manner as the plant samples, and quantitative calibration standards. Calibration standards and standard reference material samples were included at the beginning and end of the sample sets to control for drift during the analysis. Samples were introduced into an inductively coupled plasma mass spectrometer (ICP-MS) (Elan DRCe, PerkinElmer) and analyzed for Li, B, Na, Mg, P, K, Ca, Mn, Fe, Co, Ni, Cu, Zn, As, Se, Mo and Cd. All samples were normalized to calculated weights, as determined with an iterative algorithm using the best-measured elements, the weights of the seven weighed samples, and the solution concentrations, detailed at www.ionomicshub.org.

### Determination of SA content

Total SA content was quantified in leaves of five weeks old plants using a Waters Alliance HPLC system equipped with Millenium software, 2695 Separation Module, 2475 Fluorescence Detector, and 2996 Photodiode array detector. A Nova-Pack C-18 column was used with a flow rate and methanol gradient as described previously [26]. SA (Sigma; catalogue no. S-6271) was used to develop the standard curve for quantification.

### Grafting

*cpr5-2* and Col-0 seeds were germinated in the dark on 0.5× MS plates containing 1 mL L^−1^ of MS Vitamins (Caisson Laboratories, Inc.), 3 mg L^−1^ Benomyl (methyl 1-(butylcarbamoyl)-2-benzimidazolecarbamate; Sigma), 0.04 mg L^−1^ BA (6-benzylaminopurine; Sigma), 0.02 mg L^−1^ IAA (indole acetic acid; Sigma), and 12 g L^−1^ agar. Plates were held vertically and after seven days seedlings were grafted as previously described [27] and then grown for an additional seven days on plates before transfer into soil. Plants were grown for a further four weeks in soil before being analyzed for their elemental content. Plants that from a visual inspection showed adventitious roots coming from the shoot above the graft were excluded from the experiment.

### Statistical analysis

ANOVA was conducted using the software CoStat 6.2 (CoHort Software, CA, USA). Separation of means was performed using LSD test at P = 0.05 significance level.

## Results

### Mutant identification

**Figure 1:**
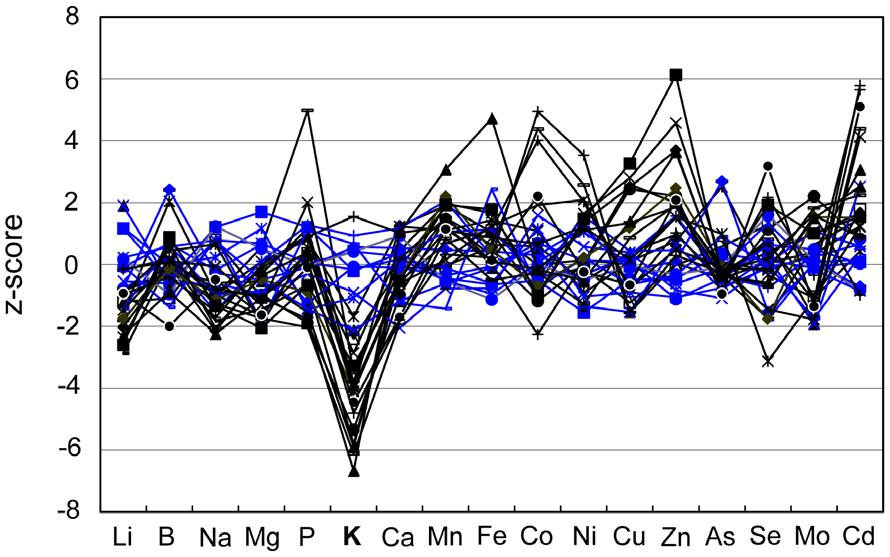
Leaf ionomic profile of *cpr5-3* (*12645*). The concentration of each element was measured in leaves of five weeks old plants of both *12645* (M3 generation) (black lines; N = 21) and wild type Col-0 (blue lines; N = 12) plants. For each element given on the abscissa the correspondent z-score value is given on the ordinate. The z-score represents the number of standard deviations of the wild-type Col-0 each plant differs from the mean of the wild-type Col-0 (raw data are available at www.ionomicshub.org, experimental tray 743).

Fast neutron mutagenized *A. thaliana* Col-0 plants were previously screened for altered leaf elemental composition [11]. In this screen *12645* was identified as a low K^+^ mutant with a reduction in leaf K^+^ of approximately 20% compared to wild-type Col-0 (raw data are available at www.ionomicshub.org, experimental tray 229) (Fig. 1). In addition, *12645* was also smaller in size than wild-type Col-0 and developed symptoms of hypersenescence in cotyledons and mature leaves. Moreover, cotyledons of mutant plants were hyponastic when seeds were germinated in soil, as well as in plates.

### Genetic analysis

To map and define the Mendelian character of the mutation, *12645* was out-crossed to L*er*-0, and F1 and F2 populations scored for mutant like plants. All F1 plants from this cross looked wild-type, while in the F2 small plants with early senescent leaves segregated with a ratio of 3:1 (wild-type to mutant phenotype), indicating that *12645* is inherited as a single recessive nuclear mutation. Small hypersenescent plants from the F2 population also showed reduced K^+^ content (38.3 µg g^−1^ dry weight) compared to those that looked wild-type (42.8 µg g^−1^ dry weight), indicating that the traits of early senescence and small size co-segregate with reduced leaf K^+^ (*P*<0.001).

**Figure 2:**
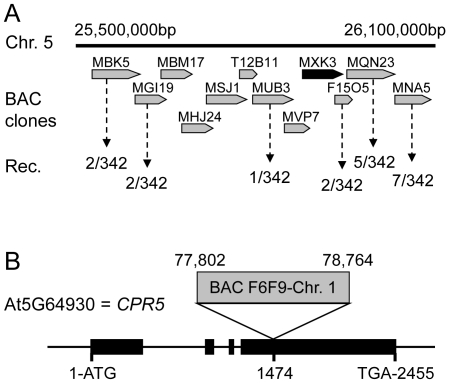
Mapping and gene structure of *cpr5-3* (*12645).* **A**. BAC clones covering the *cpr5-3* (*12645)* locus. Frequency of recombination is expressed as number of recombinant chromosomes over the total number of examined chromosomes. **B**. 972 bp insertion from BAC clone F6F9 from chromosome 1 inserted at nucleotide 1474 in the fourth exon of At5g64930 gene. Exons are shown by boxes and introns by lines.

The chromosomal position of the *12645* mutation was determined using a positional cloning approach with SSLP markers in 171 F2 plants from the outcross with L*er*-0. The recombinant population scored for markers in the region between nucleotides 25,500,000 and 26,100,000 on chromosome 5 revealed that only one plant had L*er*-0 alleles on the BAC clone MUB3 and two plants on F15O5, which placed the mutation on the BAC clone MXK3 (Fig. 2A). DNA sequencing of this region revealed that *12645* contains a 972 base pair insertion at nucleotide 1474 in the fourth exon of the At5g64930 gene (Fig. 2B). This insertion originated from nucleotides 77,802–78,764 on chromosome 1, a region that does not contain any annotated loci. *A. thaliana* mutants carrying recessive loss-of-function alleles of At5g64930 have been previously and independently identified in screens for constitutive expression of pathogen resistance, and the early appearance of hypersenescence symptoms, and named *cpr5-1* [13], *cpr5-2* [12] and *hys1* [17]. Based on the previous nomenclature we renamed *12645* as *cpr5-3*. As expected *CPR5* expression is lost in *cpr5-3* (Fig. S1A). All *cpr5* mutants (*cpr5-1*, *cpr5-2* and *cpr5-3*) were grown together, along with the wild-type Col-0 and the concentration of Li, B, Na, Mg, P, S, K, Ca, Mn, Fe, Co, Ni, Cu, Zn, As, Se, Mo, Cd in leaves of each genotype determined by ICP-MS (n = 10–22 replicate plants per genotype). Of all the elements measured only K^+^ was found to be significantly different between the wild-type Col-0 and all the *cpr5* alleles (P<0.005 after Bonferroni correction for multiple testing). Wild-type Col-0 had a leaf K^+^ concentration of 40,067±6222 µg g^−1^ dry weight, compared to 27,490±3575 µg g^−1^ dry weight for *cpr5-1*, 29,751±2616 µg g^−1^ dry weight for *cpr5-2*, and 27,324±2502 µg g^−1^ dry for *cpr5-3* (raw data are available at www.ionomicshub.org, experimental tray 1020).

### Low leaf K^+^ in *cpr5* is not dependent on SA or JA, and it is a unique trait of *cpr5* among lesion mimic mutants

**Figure 3:**
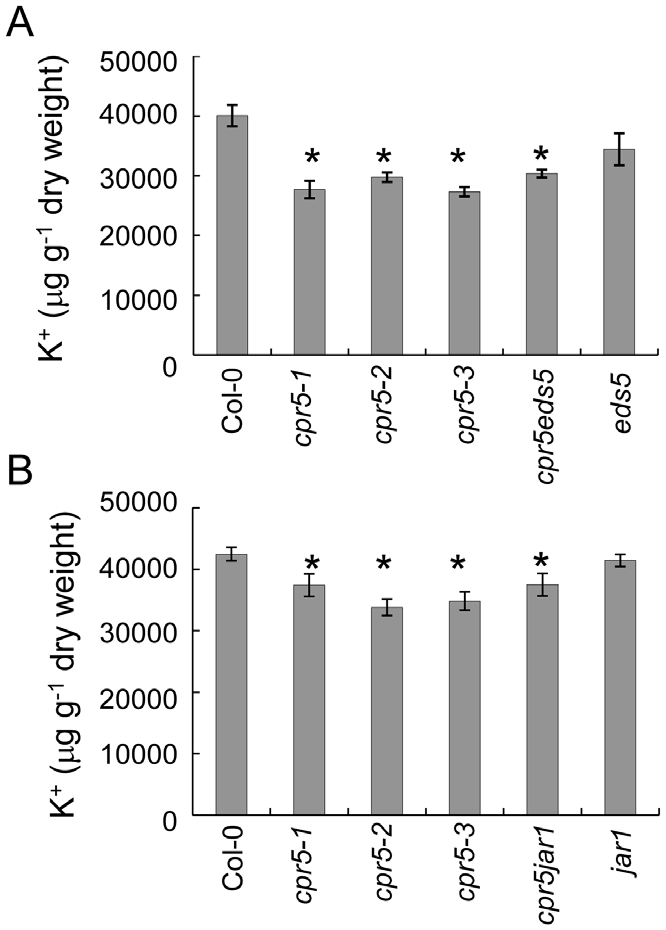
K^+^ content in salicylic acid and jasmonic acid defective mutants. K^+^ (µg g^-1^ of dry weight) was measured in leaves of five weeks old plants of wild-type Col-0, *cpr5-1*, *cpr5-2*, *cpr5-3* and (**A**) *eds5* and *cpr5eds5*, and (**B**) *jar1* and *cpr5jar1*. Wild-type Col-0 was used as a control. Values represent means of independent biological replicates (between 10 and 22 plants per genotype). Error bars represent standard errors. An asterisk (*) indicates values significantly different from the mean of the wild-type Col-0 at 99% confidence interval (raw data are available at www.ionomicshub.org; experimental trays 838, 839, 1020 and 1840).

*CPR5* acts as a negative regulator of local resistance to pathogens by partially repressing the accumulation of SA, so that *cpr5* mutants have elevated SA content [13], [14], which we also confirmed to be the case for *cpr5-3* (Fig. S1C). This raised the question, whether low leaf K^+^ in *cpr5* results from the constitutively high SA in this mutant. To address this we measured K^+^ content in three different *cpr5* alleles, in the single *eds5-1* mutant, and in the double mutant *cpr5eds5* prepared from *cpr5-1* and *eds5-1* [14]. The mutation in *eds5-1* suppresses the biosynthesis of SA and in the double mutant *cpr5eds5* the elevated SA content of *cpr5-1* is returned to the level of the wild-type [14], [28], [29]. As shown in Fig. 3A, K ^+^ content in *eds5-1* leaves is slightly reduced but not significantly different from Col-0 whereas in *cpr5eds5*, which has low SA but carries the *cpr5-1* mutation, the content of K^+^ is similar to that of single *cpr5* alleles (raw data are available at www.ionomicshub.org, experimental tray 1020).

We also followed a similar approach to determine if the low leaf K^+^ of *cpr5* is dependent on the elevated JA in this mutant [16]. The *jasmonate resistant 1* (*jar1)* mutant is unable to conjugate JA with isoleucine to form the active jasmonoyl-L-isoleucine form of JA that is required to elicit the JA response [30], and therefore this mutant is insensitive to JA [31]. Fig. 3B shows that the double mutant *cpr5jar1*, prepared from *cpr5-1* and *jar1-1* [14], and single allelic mutants *cpr5-1, cpr5-2* and *cpr5-3* all share a similar reduction of leaf K^+^ which is not observed in the single *jar1-1* mutant (raw data are available at www.ionomicshub.org; experimental tray 1840). From this we conclude that the low leaf K^+^ of *cpr5* is not dependent on JA signalling. Taken together these experiments support the conclusion that the reduced leaf K^+^ of *cpr5* is independent of both SA and JA.

**Figure 4:**
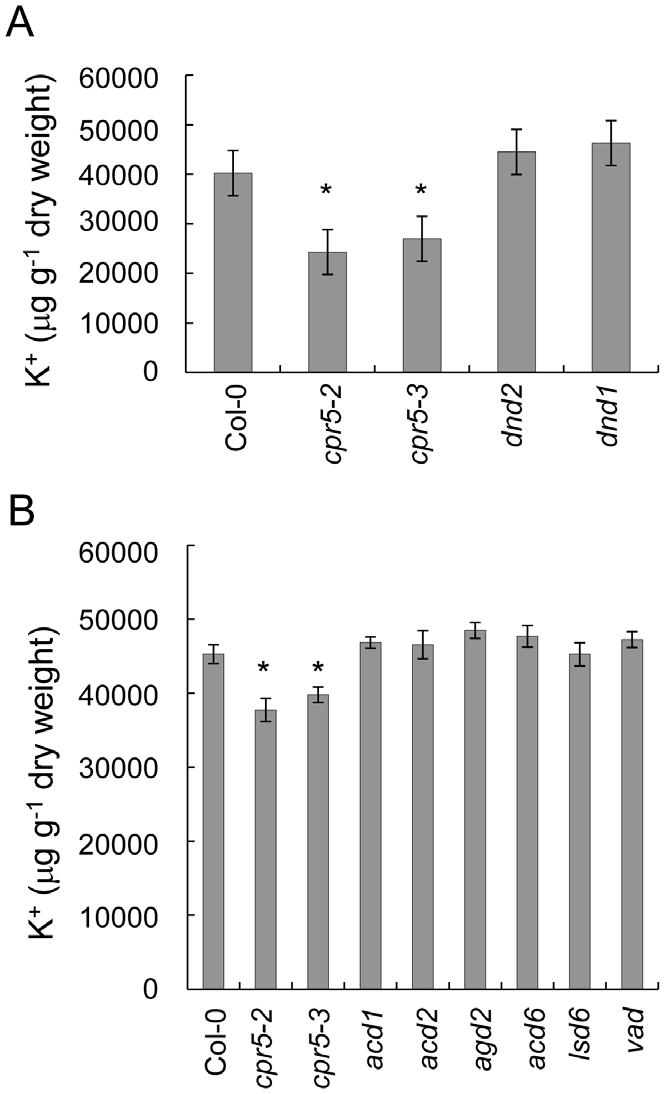
K^+^ content in lesion mimic mutants. K^+^ (µg g^−1^ of dry weight) content was measured in leaves of five weeks old plants of *cpr5-1*, *cpr5-2*, *cpr5-3* and (**A**) initiation of lesion mimic mutants *dnd1* and *dnd2*, and (**B**) initiation of lesion mimic *agd2*, *acd6*, *lsd6*, and propagation of lesion mimic mutants *acd1*, *acd2*, *vad1*. Wild-type Col-0 was used as a control. Values represent means of independent biological replicates (between 10 and 22 plants per genotype). Error bars represent standard errors. An asterisk (*) indicates values significantly different from wild-type Col-0 at 99% confidence interval (raw data are available at www.ionomicshub.org; experimental trays 1770 and 1771).

*cpr5* is easily distinguishable from wild-type plants from the presence of necrotic and chlorotic spots on the leaves, a trait which characterizes plants infected by bacteria and/or fungi, as well as mutants with a constitutively active response to pathogens. Hypothesizing that low leaf K^+^ was associated with the response to pathogens, we measured K^+^ content in leaves of lesion mimic mutants (LMM) selected from the two major classes of initiation (*dnd1, dnd2, agd2, acd6, lsd6*) and propagation (*acd1, acd2, vad1*) of lesion mutants [32]. None of the lesion mimic mutants tested showed reduced leaf K^+^ (Fig. 4A, B). From this we conclude that the low leaf K^+^ observed in *cpr5* is not a result of the presence of lesions.

### Low leaf K^+^ in *cpr5* is primarily driven by the shoot but roots also play a role

**Figure 5:**
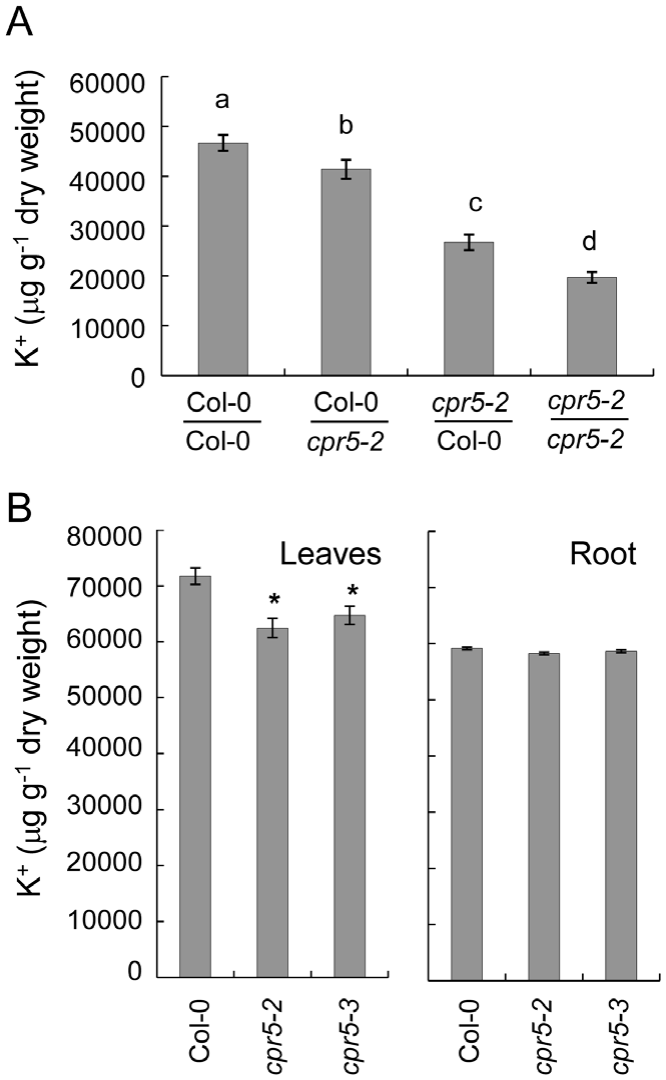
K^+^ content in *cpr5* root and leaves and in *cpr5* grafted plants. **A**. K^+^ (µg g^-1^ of dry weight) in leaves of grafted plants transferred to soil after grafting and grown for four weeks. Values represent means of independent measurements performed on 19 to 32 different plants per graft combination. Error bars show standard errors. Bars with different letters are significantly different at *P* = 0.05 level (LSD test). Col-0/Col-0, self-grafted wild-type Col-0; Col-0/*cpr5-2*, wild-type Col-0 shoot grafted on *cpr5-2* root; *cpr5-2*/Col-0, *cpr5-2* shoot grafted on wild-type Col-0 root; *cpr5-2/cpr5-2*, self-grafted *cpr5-2*. **B**. K^+^ (µg g^−1^ of dry weight) in leaves and roots of wild-type Col-0, *cpr5-2* and *cpr5-3* plants grown for two weeks on solidified 1/20^th^ MS medium supplemented with 20 mM of K^+^. Data represents the mean of 10 measurements of a pool of 10 to 15 shoots or roots per genotype grown on 15 different plates. Error bars represent standard errors. An asterisk (*) indicates values significantly different from the mean of wild-type Col-0 at *P* = 0.005.

Low K^+^ in *cpr5* leaves could be caused by reduced uptake from roots, impaired root-to- shoot translocation, or enhanced shoot-to-root circulation through the phloem. *CPR5* is equally expressed in both roots and leaves (Fig. S1B) making it possible that *CPR5* could contribute to K^+^ homeostasis in either organ. Therefore, we performed reciprocal grafting of *cpr5* and wild-type Col-0 to determine in which tissue *cpr5* exerts its influence. Shoots of *cpr5-2* were grafted onto wild-type Col-0 roots and vice versa, grafted plants allowed to grow for four weeks in soil and the K^+^ content measured in leaves. When *cpr5-2* shoots were grafted on wild-type Col-0 roots we observed the K^+^ content in leaves to be significantly reduced by 43% compared to self-grafted Col-0 (Fig. 5A). Moreover, plants with *cpr5-2* shoot and wild-type Col-0 root also retained the hypersenescence phenotype of chlorotic and necrotic spots observed on leaves of non-grafted *cpr5-2* plants. In contrast, plants with wild-type Col-0 shoots grafted on *cpr5-2* roots showed only an 11% reduction in leaf K^+^ and were indistinguishable from Col-0 in respect to symptoms of hypersenescence. Interestingly, self-grafted *cpr5-2* plants showed the lowest leaf K^+^ of all the grafting types tested, with a reduction in leaf K of 58%. These results suggest that loss-of-function of *CPR5* in shoots plays a primary role in the reduced leaf K^+^ phenotype of *cpr5*, but loss-of-function of *CPR5* in roots also exerts a lesser yet significant influence.

To further understand the role of roots versus shoots in the low leaf K^+^ phenotype of *cpr5* we measured the K^+^ content of shoot and root tissue of plants grown on solidified MS medium in plates. This experiment revealed no significant difference in the root concentration of K^+^ between *cpr5* and Col-0, while *cpr5* shoots retained the low K^+^ phenotype observed in plants grown in soil (Fig. 5B).

### Expression of *Cyclic Nucleotide Gated Channels* is elevated in shoots of *cpr5*

**Figure 6:**
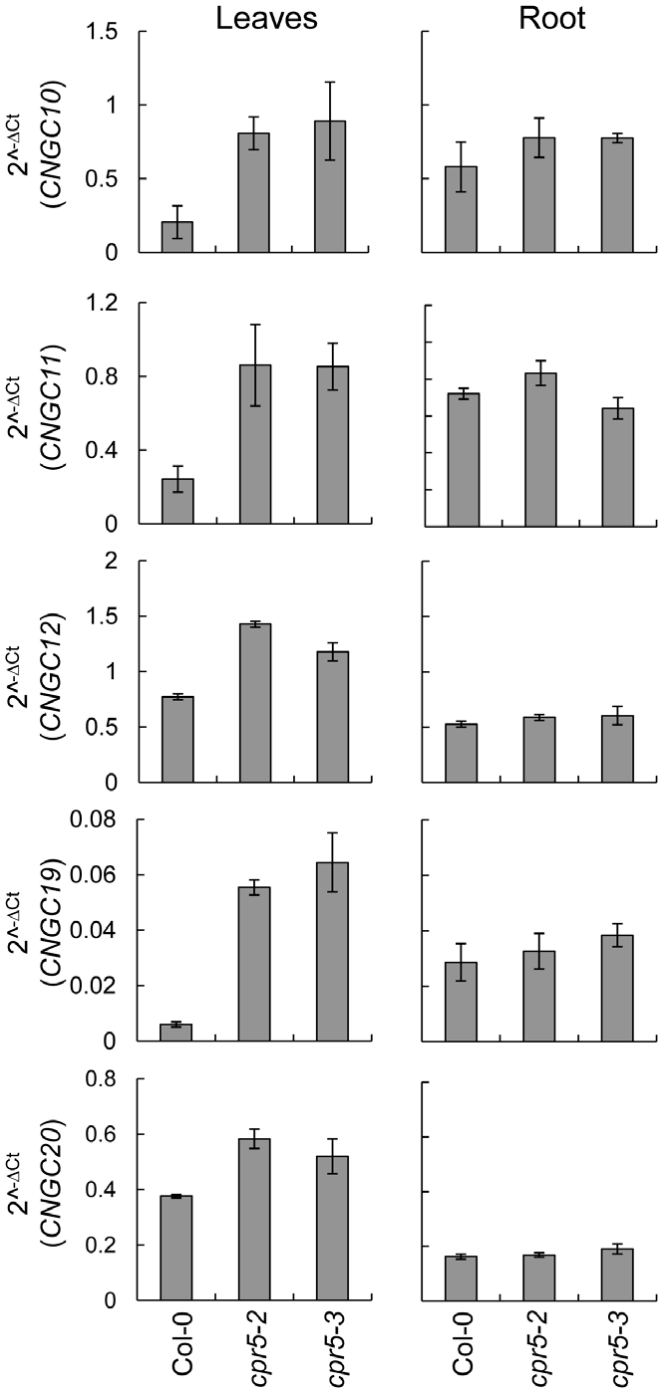
Expression of *CNGCs* in *cpr5*. Steady state levels of *CNGC10, CNGC11, CNGC12, CNGC19, CNGC20* mRNA in leaves and roots of *cpr5-2*, *cpr5-3* and wild-type Col-0 evaluated by qRT-PCR. Seedlings were germinated and grown on 1/20^th^ MS medium supplemented with 20 mM KCl. After two-week of growth shoot and root tissue was harvested and RNA extract for qRT-PCR. *PP2A* (At1g13320) was used as an endogenous reference gene for normalization across samples, and data presented as 2^−Δ*C*t^. Error bars represent standard deviations calculated following [29].

Based on the importance of the shoot in driving the reduced leaf K^+^ in *cpr5* we investigated expression of *Cyclic Nucleotide Gated Channels* (*CNGCs)* that may be directly or indirectly involved in K^+^ efflux during the response to pathogens in *A. thaliana*. An initial survey of transcriptional data publically available in experiment 175 on Genevestigator [33] revealed that the expression of numerous *CNGCs* was up-regulated in *cpr5* leaves. We confirmed the differences initially observed in the database using qRT-PCR, and performed similar measurements in root tissue. Our analysis revealed that steady state levels of *CNGC10*, *CNGC11*, *CNGC12*, *CNGC19* and *CNGC20* mRNA are all elevated in leaves of both *cpr5-2* and *cpr5-3* compared to wild-type Col-0 (Fig. 6). Analogous measurements performed in roots did not revealed any substantial differences from wild-type Col-0 for the *cpr5* mutants (Fig. 6).

### Expression of *HAK5* encoding a high affinity K^+^ transporter is reduced in roots of *cpr5*

**Figure 7:**
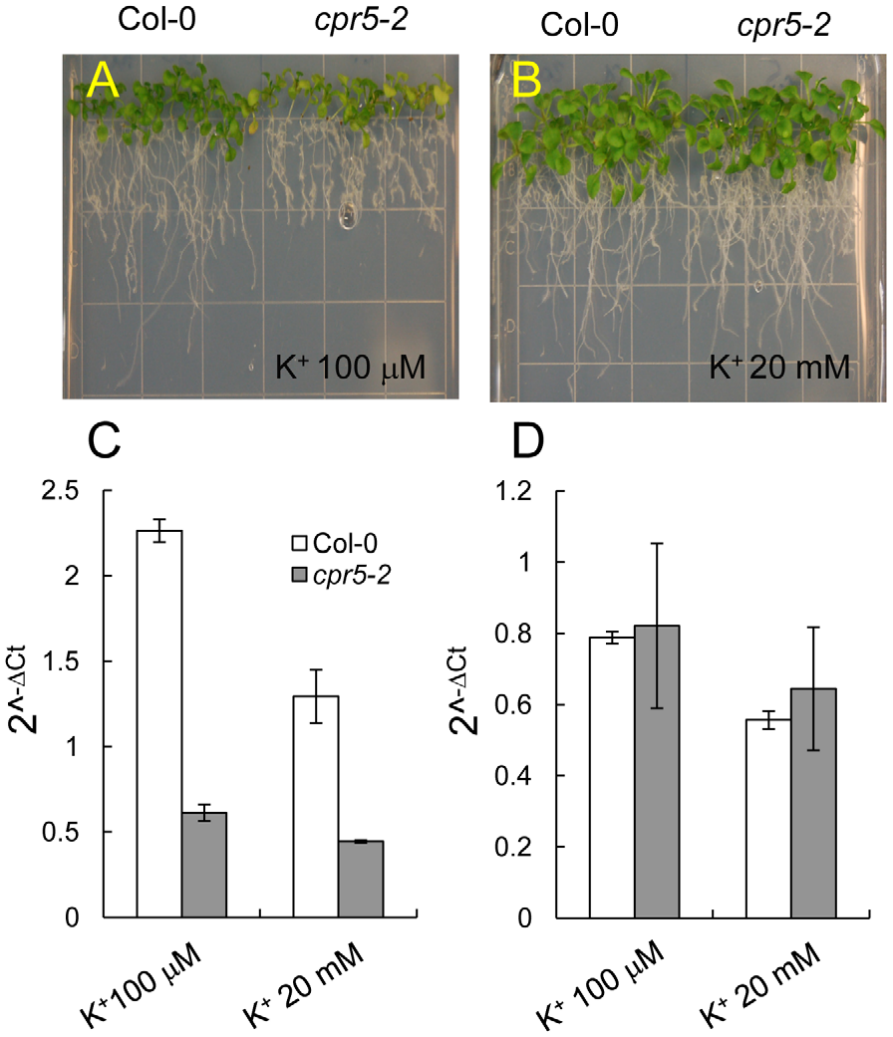
Expression of *HAK5* and *AKT1* in roots of *cpr5*. Seeds were germinated on solidified 0.5× MS salts, and after five days seedlings transferred to solidified 1/20^th^ MS medium supplemented with either 100 µM (**A**) or 20 mM KCl (**B**). Pictures were taken after two weeks of growth. Steady state levels of *HAK5* (**C**) and *AKT1* (**D**) mRNA were quantified using qRT-PCR in roots of wild-type Col-0 (white bars) and *cpr5-2* (gray bar). RNA was extracted from roots of three independent samples generated from between 25 and 30 plants per plates. *UBQ10* (At4g05320) was used as an endogenous reference gene for normalization across the samples, and data presented as 2^−Δ*C*t^. Error bars represent standard deviations calculated following [29].

The K^+^ transporter *HAK5* is known to contributes to K^+^ uptake in *A. thaliana* primarily at low K^+^ supply (0–0.25 mM) [23], [34]–[37], whereas the K^+^ channel *AKT1* [38], [39] contributes to K^+^ uptake at both low and intermediate K^+^ supply (0.01–5 mM) [35]–[37], [40]. Above 5 mM external K^+^ the transport processes involved in K^+^ uptake are currently undefined [36]. Given the importance of both *HAK5* and *AKT1* in K^+^ uptake in *A. thaliana* we used qRT-PCR to quantify the steady state levels of *HAK5* and *AKT1* mRNA in *cpr5* roots to test if altered expression of these genes may be involved in the low leaf K^+^ phenotype we observe in *cpr5*. Steady state levels of *AKT1* mRNA in *cpr5* where found to be the same as wild-type Col-0 after growth on medium supplemented with either high (20 mM) or low (100 µM) K^+^ (Fig. 7C). A slight increase in *AKT1* mRNA was observed in wild-type Col-0 plants grown on medium supplemented with 100 µM K^+^ compared to 20 mM K^+^. Enhanced expression of *AKT1* was not previously observed [41], though this is possibly due to the fact that the previous authors used RT-PCR to determine expression of *AKT1*. Interestingly, steady state levels of *HAK5* mRNA were observed to be reduced in *cpr5* compared to wild-type Col-0 grown in medium with either high and low K^+^ supply (Fig. 7B). Further, the increase in *HAK5* mRNA observed in wild-type Col-0 grown on medium containing 100 µM K^+^ was essentially abolished in *cpr5* (Fig. 7B). Interestingly, root growth of *cpr5* on medium supplemented with 100 µM K^+^ was found to be reduced compared to wild-type (Fig. 7A). However, in medium supplemented with 20 mM K^+^ growth of *cpr5* and wild-type was similar (Fig. 7A).

**Figure 8:**
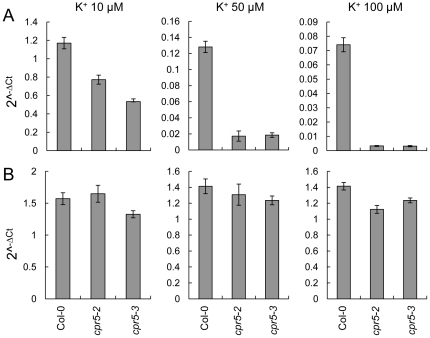
Expression of *HAK5* and *AKT1* in roots of *cpr5* grown in the absence of NH_4_^+^. Wild-type Col-0, *cpr5-2* and *cpr5-3* seedlings were grown on solidified defined medium without NH_4_^+^ and supplemented with different concentrations of KCl (10, 50 and 100 µM). RNA was extracted from roots of two week old plants and steady state levels of *HAK5* (**A**) and *AKT1* (**B**) quantified using qRT-PCR. Data represents the mean of four biological replicates each composed of a pool of 5 to 10 plants. *PP2A* (*At1g13320*) was used as an endogenous reference gene for normalization across samples, and data presented as 2^−Δ*C*t^. Error bars represent standard deviations calculated following [29].

To confirm and extend these results we performed a more detailed dose response experiment with solidified medium supplemented with 10, 50, and 100 µM KCl lacking NH_4_^+^ given that NH_4_^+^ is known to suppress activity [38], [39] and expression [23] of *HAK5*. Pure agarose was used to solidify the growth medium to avoid any extra K^+^ supply [42]. In wild-type Col-0 root steady state levels of *HAK5* mRNA were observed to increase as external K^+^ was reduced (Fig. 8A), as expected [34]. In roots of *cpr5*-2 and *cpr5-3* steady state levels of *HAK5* mRNA were also increased as the external K^+^ concentration was reduced (Fig. 8A). However, steady state levels of *HAK5* mRNA in *cpr5-2 and cpr5-3* were consistently lower then wild-type Col-0 at 10, 50 and 100 µM K^+^ in the growth medium (Fig. 8A). In agreement with our previous observations no consistent differences in expression of *AKT1* between wild-type Col-0 and *cpr5* were observed (Fig. 8B).

## Discussion

The data presented here establishes that the wild-type allele of *CPR5* is required to maintain normal K^+^ homeostasis in *A. thaliana* Col-0. We observed that loss-of-function alleles of *CPR5* show a consistent and specific reduction in leaf K^+^ of 10–30% in plants grown in both soil or on solidified MS medium with high K^+^ supply. Further, this defect leads to reduced growth at low K^+^ supply. Genetic analysis confirmed that this reduction in leaf K^+^ is not dependent on the elevated SA or JA known to occur in *cpr5*. Further, genetic analysis confirmed that reduced leaf K^+^ is not a feature of lesion mimic mutants in general. Through reciprocal grafting we establish that the reduced leaf K^+^ observed in *cpr5* is primarily driven by the shoot (74%), with the root playing a significant but smaller role (19%). Interestingly, the presence of the *cpr5* allele in both roots and shoots is required to produce the full low leaf K^+^ phenotype, suggesting that feedback between both organs is needed. In investigating the factors that in *cpr5* cause the reduction of leaf K^+^, we surveyed the experiment AT-175 performed on *cpr5* shoots (www.genevestigator.ethz.ch) and this revealed that genes belonging to the *CNGC* family are highly expressed in *cpr5*. Using qRT-PCR we confirmed that *CNGC10*, *CNGC11*, *CNGC12*, *CNGC19*, *CNGC20* are indeed highly expressed in leaves, but not roots, of *cpr5*. The expression of *CNGC1* and *CNGC13* which showed a milder increase in the AT-175 array did not show any difference when expression was analyzed by qRT-PCR. *CNCG2* and *CNGC4*, null mutants of which (*dnd1* and *dnd2)* have enhanced disease resistance, also did not show any misregulation when analyzed by qRT-PCR. In the interaction between plant and pathogens the recognition of factors of avirulence by the host plant induces fluxes of Ca^2+^ that initiate the immune response and leads to enhanced K^+^ efflux [10]. *CNGCs* are believed to be the channels that deliver the Ca^2+^ signal required for pathogen recognition [43], and *dnd1*, *dnd2* and the gain of function chimeric *CNGC11/12* mutant *cpr22* strongly support this conclusion [44]–[50]. Moreover, heterologous expression of specific *CNGCs* provides direct evidence of Ca^2+^ transport activity [51], [52]. It is therefore possible that in *cpr5* the constitutively high level of expression of *CNGCs* we observe leads to a persistent activation of this Ca^2+^ initiated signalling cascade that in turn leads to constitutively enhanced K^+^ efflux [10]. Such an enhanced K^+^ efflux could increase shoot-to-root K^+^ export, and may explain the leaf-driven portion of the reduced leaf K^+^ observed in *cpr5*. The transporters/channels involved in this K^+^ efflux are currently unknown. However, these is evidence that *CNGC10* transports K^+^ [53], and steady state levels of *CNGC10* mRNA are elevated in *cpr5*, making it possible that CNGC10 could play a role in the proposed enhanced K^+^ efflux and shoot-to-root export in *cpr5*.

*HAK5* encodes the primary high affinity root K^+^ transporter in *A. thaliana* [23], [34]–[37], and its expression in wild-type Col-0 roots is induced at low K^+^ supply [34]. Significantly, *cpr5* roots show a consistent reduction in the steady state levels of *HAK5* mRNA under both high and low K^+^ supply. Though *HAK5* primarily plays a role in K^+^ uptake at low K^+^ supply, it is possible that the reduced steady state levels of *HAK5* mRNA in *cpr5* roots we observe even at high K^+^ supply is responsible for the small (18%) but significant contribution of roots to the reduced leaf K^+^ phenotype of *cpr5*. It is also interesting to speculate that expression of *HAK5* in *cpr5* roots is suppressed by an increased flux of K^+^ arriving in the roots from the shoots driven by elevated expression of *CNGCs* in shoots.

Based on sequence analysis *CPR5* encode a membrane protein with five transmembrane domains and a nuclear localization signal (NLS). *CPR5* appears to localize to the nucleus [54] where it has been proposed to be targeted to the inner nuclear membrane [19], [54]. It has been further suggested that proteolytic cleavage can release the nucleosolic domain of *CPR5* from the membrane to allow its participation in transcriptional processes [19], [54]. Recent studies have identified the transcription factors *TPR1* and *EDS1* that direct repression of expression of *CNGC2* and *CNGC4* [55], [56], and we speculate that *CPR5* may play a similar role in the nucleus to negatively regulate the expression of the various *CNGCs* affected on the *cpr5* mutant.

In summary, we have identified a new low leaf K^+^ phenotype for *cpr5* that is primarily driven by the shoot, and is independent of the elevated levels of SA and JA found in this mutant. We suggest that the reduced leaf K^+^ of *cpr5* is caused by the elevated expression of various *CNGCs* in shoots and the reduced expression of *HAK5* in roots, driving both an enhanced K^+^ export from shoots and a reduced K^+^ uptake in roots. Our observation of altered K^+^ homeostasis in *cpr5* may also be an important piece of evidence linking the function of *CPR5* as a negative regulator of local defence responses to pathogens with the role K^+^ efflux plays in these responses; likely through the direct or indirect regulation of *CNGC*s by *CPR5*.

## Supporting Information

Figure S1 ***CPR5*** **expression and salicylic acid (SA) levels in** ***A. thaliana*** **leaves and roots of wild-type Col-0 and** ***cpr5.*** **A**. Steady state levels of *CPR5* mRNA in wild-type Col-0 and *cpr5-3* quantified using qRT-PCR. RNA was extracted from leaves of five-week old plants grown in soil. Data represents the mean of measurements of four independent biological replicates. Each biological replicate consisted of 2-3 leaves from individual plants. Errors bars represent standard deviation. **B**. Steady state levels of *CPR5* mRNA in root and shoots of wild-type Col-0 quantified using qRT-PCR. RNA was extracted from shoots and roots of two week old wild-type Col-0 plants grown on 0.5× MS media solidified with 1% agar (w/v). Values represent mean of measurements from at least three independent replicates. Errors bars represent standard deviation. Steady state mRNA levels (**A** & **B**) are presented as 2^−Δ*C*t^. *UBQ10* (At4g05320) was used as an endogenous reference gene for normalization across samples. **C**. SA content (mg g^−1^ of fresh weight) in leaves of wild-type Col-0, *cpr5-2* and *cpr5-3.* Data represents the mean of three independent leaf samples harvested from individual plants grown in soil for five weeks. Error bars represent the standard error.
(TIF)
