# Supplementary figures and images for: Loss-of-Function of Constitutive Expresser of Pathogenesis Related Genes5 Affects Potassium Homeostasis in Arabidopsis thaliana
Source: PLoS One. 2011 Oct 27;6(10):e26360. doi: 10.1371/journal.pone.0026360 (PMC3203115; doi:10.1371/journal.pone.0026360)

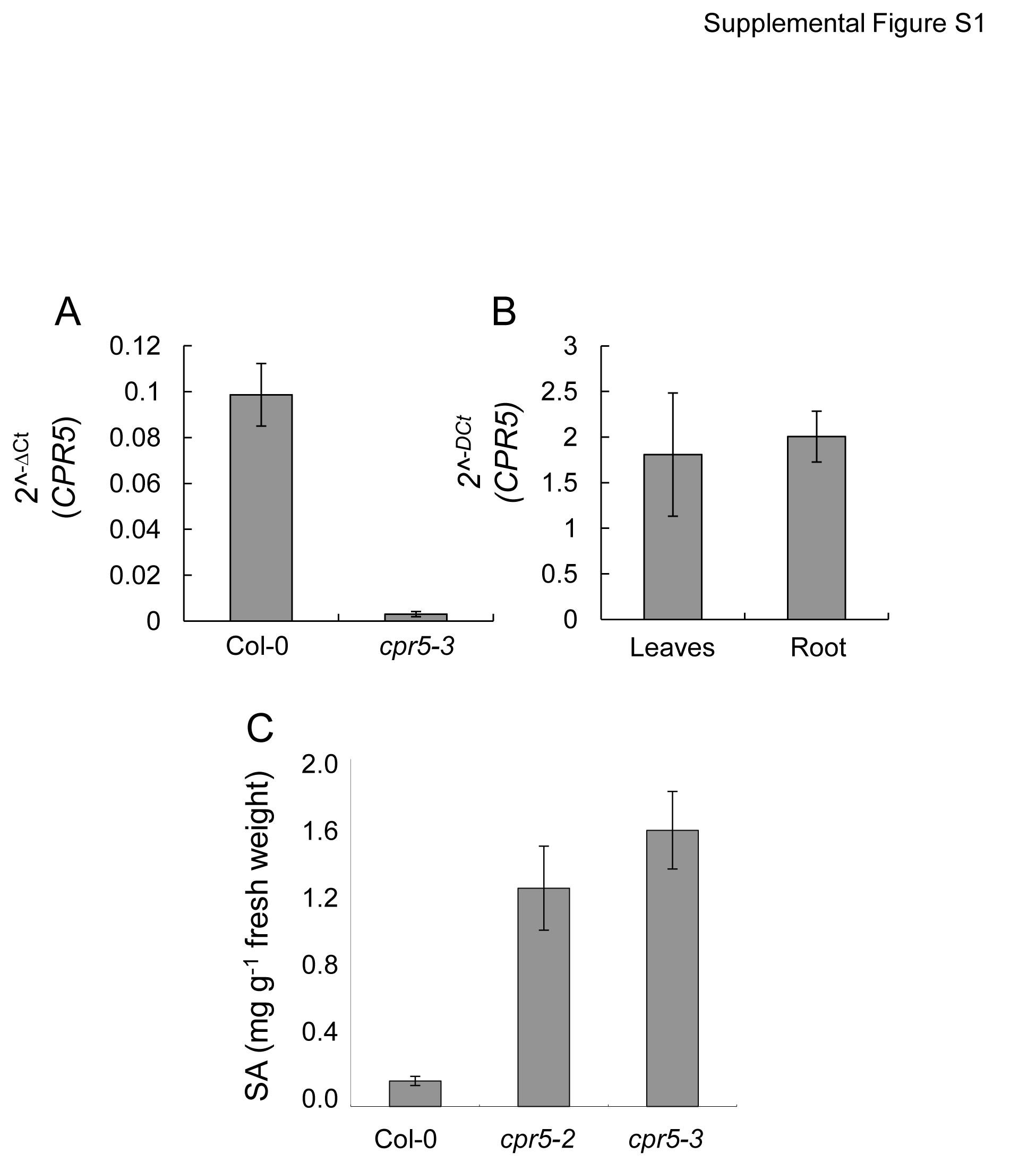

Supplement: Figure S1 — CPR5 expression and salicylic acid (SA) levels in A. thaliana leaves and roots of wild-type Col-0 and cpr5. A. Steady state levels of CPR5 mRNA in wild-type Col-0 and cpr5-3 quantified using qRT-PCR. RNA was extracted from leaves of five-week old plants grown in soil. Data represents the mean of measurements of four independent biological replicates. Each biological replicate consisted of 2-3 leaves from individual plants. Errors bars represent standard deviation. B. Steady state levels of CPR5 mRNA in root and shoots of wild-type Col-0 quantified using qRT-PCR. RNA was extracted from shoots and roots of two week old wild-type Col-0 plants grown on 0.5× MS media solidified with 1% agar (w/v). Values represent mean of measurements from at least three independent replicates. Errors bars represent standard deviation. Steady state mRNA levels (A & B) are presented as 2−ΔCt. UBQ10 (At4g05320) was used as an endogenous reference gene for normalization across samples. C. SA content (mg g−1 of fresh weight) in leaves of wild-type Col-0, cpr5-2 and cpr5-3. Data represents the mean of three independent leaf samples harvested from individual plants grown in soil for five weeks. Error bars represent the standard error. (TIF) [file pone.0026360.s001.tif]
